# The impact of Wilson disease on myocardial tissue and function: a cardiovascular magnetic resonance study

**DOI:** 10.1186/s12968-021-00760-1

**Published:** 2021-06-24

**Authors:** Janek Salatzki, Isabelle Mohr, Jannick Heins, Mert H. Cerci, Andreas Ochs, Oliver Paul, Johannes Riffel, Florian André, Kristóf Hirschberg, Matthias Müller-Hennessen, Evangelos Giannitsis, Matthias G. Friedrich, Uta Merle, Karl Heinz Weiss, Hugo A. Katus, Marco Ochs

**Affiliations:** 1 Department of Cardiology, Angiology and Pneumology, Heidelberg University Hospital, Heidelberg, Germany; 2 DZHK (German Centre for Cardiovascular Research), Partner site Heidelberg, Heidelberg, Germany; 3 Department of Gastroenterology, Heidelberg University Hospital, Heidelberg, Germany; 4 Semmelweis University Heart and Vascular Center, Budapest, Hungary; 5 Division of Cardiology, Departments of Medicine and Diagnostic Radiology, Mc-Gill University Health Centre, Montreal, Canada; 6 Department of Internal Medicine, Salem Medical Center, Heidelberg, Germany

**Keywords:** Wilson Disease, Myocardial fibrosis, Strain, Extracellular volume fraction

## Abstract

**Background:**

Systemic effects of altered serum copper processing in Wilson Disease (WD) might induce myocardial copper deposition and consequently myocardial dysfunction and structural remodeling. This study sought to investigate the prevalence, manifestation and predictors of myocardial tissue abnormalities in WD patients.

**Methods:**

We prospectively enrolled WD patients and an age-matched group of healthy individuals. We applied cardiovascular magnetic resonance (CMR) to analyze myocardial function, strain, and tissue characteristics. A subgroup analysis of WD patients with predominant neurological (WD-neuro^**+**^) or hepatic manifestation only (WD-neuro^**−**^) was performed.

**Results:**

Seventy-six patients (37 years (27–49), 47% women) with known WD and 76 age-matched healthy control subjects were studied. The prevalence of atrial fibrillation in WD patients was 5% and the prevalence of symptomatic heart failure was 2.6%. Compared to healthy controls, patients with WD had a reduced left ventricular global circumferential strain (LV-GCS), and also showed abnormalities consistent with global and regional myocardial fibrosis. WD-neuro^**+**^ patients presented with more severe structural remodeling and functional impairment when compared to WD-neuro^**−**^ patients.

**Conclusions:**

In a large cohort, WD was not linked to a distinct cardiac phenotype except CMR indexes of myocardial fibrosis. More research is warranted to assess the prognostic implications of these findings. *Trial registration:* This trial is registered at the local institutional ethics committee (S-188/2018).

**Supplementary Information:**

The online version contains supplementary material available at 10.1186/s12968-021-00760-1.

## Background

Wilson disease (WD) is a rare autosomal recessive disorder caused by a genetic defect in ATP7B resulting in limited excretion of excess copper into the bile [1–3]. Pathological copper accumulation occurs in the entire body, with the liver and the brain being primarily affected [4]. The pathological copper accumulation may induce toxic injury, including mitochondrial dysfunction [5] or apoptosis [6]. Exhausted hepatic copper storage capacity causes copper release into the bloodstream thus affecting other organs, in particular the brain [7]. The neurotoxicity of copper primarily damages astrocytes, leading to a regional destruction of the blood–brain barrier with subsequent neurological symptoms [8, 9].

Whether copper accumulation in WD induces toxic effects in cardiomyocytes and the clinical consequences of these myocardial accumulations are poorly understood. Previous studies have mainly reported mild cardiac abnormalities including concentric hypertrophy, impaired left ventricular (LV) relaxation and minor electrocardiographic changes, mainly reported in the times before appropriate treatment was available [10–13]. Grandis et al. demonstrated in a longitudinal cohort study a higher incidence of heart failure (HF) and atrial fibrillation in WD patients, indicating a potential adverse effect of copper on the heart [14].

Cardiovascular magnetic resonance (CMR) allows for a non-invasive tissue characterization, specifically visualizing edema, fibrosis, and pathological infiltrations [15]. CMR has been successfully used for diagnosis and therapy monitoring in other storage diseases such as myocardial iron overload and amyloidosis [16, 17]. Survival in patients with thalassemia major improved significantly after the introduction of CMR for identifying myocardial iron accumulation [18]. Imaging regional fibrosis using CMR late gadolinium enhancement (LGE) and quantitative measurement of extracellular volume (ECV) provide independent prognostic markers in cardiac amyloidosis [19].

The purpose of this study was to evaluate the prevalence and spectrum of myocardial manifestations in WD patients.

## Methods

### Study population and design

**Fig. 1:**
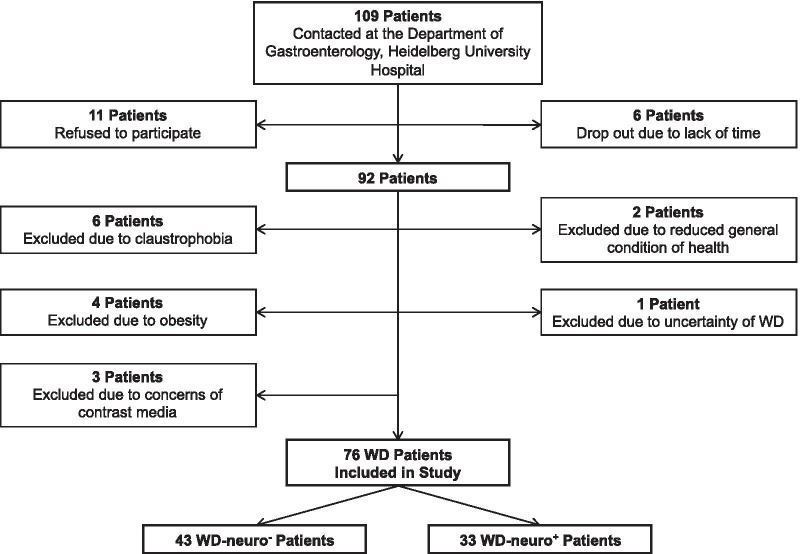
Flowchart of patients inclusion. Flowchart of Wilson Disease (WD) included in study. WD-neuro−—Wilson Disease with primarily hepatic manifestation and *without* neurological symptoms, WD-neuro + —Wilson Disease* with* neurological symptoms

Between May 2018 and December 2019, we prospectively enrolled WD patients presenting to the outpatient WD clinic of the Heidelberg University Hospital (Fig. 1). The study was approved by the local institutional ethics committee in accordance with the Declaration of Helsinki (S-188/2018) and all subjects gave written informed consent. WD patients were divided in two groups: Patients with a primarily hepatic involvement (WD-neuro^**−**^) and patients with at least one neurological symptom (WD-neuro^**+**^). An electrocardiogram (ECG) was performed in all WD patients. Information on cardiac symptoms (shortness of breath at rest or during exercise, chest pain, peripheral edemas, palpitations, dizziness, loss of consciousness), cardiovascular risk factors (arterial hypertension, history of smoking, family history of cardiovascular disease, diabetes mellitus, hypercholesterinemia), history of atrial fibrillation and HF, impaired mobility, and regular medications was collected using a detailed questionnaire and medical charts. New York Heart Association (NYHA) class was accessed by questioning patients about shortness of breath at rest or on exertion. Leipzig score of all patients was calculated, which is a diagnostic scoring system for WD with good accuracy [20, 21]. Additionally, we calculated the Unified Wilson´s Disease Rating Scale (UWDRS), which describes neurological signs and their severity [22].

### Selection of controls

We also recruited individuals free of cardiovascular disease as controls. They were matched for age, sex, body surface area (BSA), and cardiovascular risk factors to our WD cohort. This was approved by our local institutional ethics committee in accordance with the Declaration of Helsinki (S-101/2019 and S-259/2016). The requirement for individual informed consent was waived by our local institutional ethics committee.

### Study protocol

CMR was performed in a supine position a 1.5 T CMR system (Ingenia™, Philips Healthcare, Best, The Netherlands), with a commercial cardiac phased-array receiver coil as previously described [23, 24]. Details of acquisition and post-processing are available in the Additional file 1.

Native myocardial T1 maps were calculated from Modified Look-Locker Inversion recovery (MOLLI, 5 s(3 s)3 s scheme) images acquired before and 15 min after contrast agent injection [25]. T2 mapping was performed using a multi-echo gradient-spin-echo (GraSE) sequence in the same LV short-axis position as T1 mapping. T2 GraSE has been previously shown to have a good correlation with myocardial water content [26, 27]. Myocardial T2* was quantified from cardiac-gated, spoiled gradient-echo images.

(LGE images were acquired 10 min after administration of 0.14 mmol/kg of gadobutol (Gadovist, Schering, Berlin, Germany) and analysis was performed as previously described [28]. Blood samples for the determination of hematocrit (HCT) were taken on the same day before CMR. ECV was created according to the following formula:

$${\text{ECV}}\left( \% \right) \, = \, \left( {{1} - {\text{HCT}}} \right) \, * \, \left( {\left( {{\text{T1}}_{{{\text{myocardium post}} - {\text{contrast}}}} - {\text{ T1}}_{{{\text{myocardium pre}} - {\text{contrast}}}} } \right) \, / \, \left( {{\text{T1}}_{{{\text{blood post}} - {\text{contrast}}}} - {\text{ T1}}_{{{\text{blood pre}} - {\text{contrast}}}} } \right)} \right)$$

As previously described [29]. Details of mapping and LGE acquisition are available in the Additional file 1.

For strain imaging, we used a real-time variant of Strain-ENCoded (SENC) CMR imaging called fSENC, a single-shot sequence [30]. The resulting images were analyzed offline using dedicated software (MyoStrain™; Myocardial Solutions, Inc., Morrisville, North Carolina, USA). Global circumferential strain (GCS) was measured from three long-axis views and the global longitudinal strain (GLS) from the three short-axis images. Endo- and epicardial borders were drawn at the end-systolic cardiac phase. Details of the fSENC technique are provided in the Additional file 1.

### Statistical analysis

Statistical analysis was performed using SPSS (version 24.0, Statistical Package for the Social Sciences, International Business Machines, Inc., Armonk, New York, USA) and MedCalc (version 15.7, MedCalc Software, Mariakerke, Belgium), with p < 0.05 taken to indicate statistical significance for all statistical tests. Continuous and normal distributed variables (Kolmogorov–Smirnov test, p ≥ 0.05) were expressed as mean ± standard deviation. Differences between two unrelated groups for continuous variables were tested using the independent t-test. Continuous variables without normal distribution were stated as median and interquartile range (IQR), group differences were tested using the nonparametric Mann–Whitney U test between unrelated groups. Categorical variables of independent samples were compared using chi-squared test. Pearson correlation was used to assess linear relationships between variables. All significant values of independent variables were assessed against the Benjamini–Hochberg Procedure for controlling for false discovery rates [31].

## Results

### Patient characteristics

Characteristics of WD patients and matched healthy helthy controls are presented in Table 1. Seventy-six WD patients (37 years, (IQR 27–49); 47% women) were enrolled.

**Table 1:** Patient characteristics of patients with Wilson Disease

Patient characteristics	WD (n = 76)	Controls (n = 76)	p	WD- neuro^−^ (n = 43)	WD-neuro^+^ (n = 33)	p
Age (years)	37 (27–49)	36 (27–51)	0.98	37 (26–49)	35 (27.5–50)	0.82
Female (n)	36 (47%)	36 (47%)	1.00	24 (56%)	12 (36%)	0.09
BSA (m^2^)	1.93 ± 0.25	1.92 ± 0.22	0.34	1.90 ± 0.24	1.96 ± 0.26	0.30
BMI (kg/m^2^)	24 ± 4	24 ± 3	0.20	24 ± 3	25 ± 4	0.48
Heart rate (bpm)	70 ± 13	68 ± 11	0.46	72 ± 13	66 ± 13	0.06
Systolic blood pressure (mmHg)	120 ± 12	122 ± 12	0.43	121 ± 13	119 ± 10	0.65
Diastolic blood pressure (mmHg)	72 ± 13	69 ± 6	0.24	71 ± 14	75 ± 11	0.28
Hypertension	11%	14%	0.61	5%	18%	0.07
Hypercholesterolemia	11%	9%	0.67	10%	13%	0.71
Diabetes mellitus	3%	0%	0.27	2%	3%	0.85
History of smoking	23%	19%	0.62	19%	31%	0.21
Family history of CAD	12%	26%	0.05	12%	13%	0.91
Atrial fibrillation	5%	0%	< 0.05	7%	3%	0.45
Heart failure	2.6%	0%	0.31	0%	6%	0.10

Patient Characteristics of Wilson Disease (WD) patients compared to Controls and WD patients without (WD-neuro^**−**^) and with neurological symptoms (WD-neuro^**+**^): BSA, body surface area; BMI, body mass index; bpm, beats per minute; CAD, coronary artery disease. Values are mean ± SD, median (interquartile range) or n (%). Differences between groups were calculated using t-test, Mann–Whitney U test or chi-squared test

The prevalence of atrial fibrillation in WD patients was 5% (Table 1). Thirty-three WD patients suffered of neurological symptoms (WD-neuro^**+**^), while 43 did not have pathological neurological tendency, but had a primarily hepatic manifestation (WD-neuro^**−**^). The WD-neuro^**+**^ subgroup showed a non-significant trend for male sex and a higher prevalence of hypertension (p = 0.09 and 0.07 respectively) (Table 1). The median of the total Leipzig score was 5 (IQR 4–7) with a range of 4 to 11. WD-neuro^**+**^ patients had a significantly higher Leipzig score compared to WD-neuro^**−**^ patients (WD-neuro^**+**^ = 6 (IQR 5–8); WD-neuro^**−**^ = 4 (IQR 4–7); p < 0.01).

### Clinical presentation

Twelve percent of patients with WD complained of dyspnea at rest or on exertion. The median NYHA class was 1 (1–1). Additionally, a relatively large proportion of WD patients reported dizziness (24%) and loss of consciousness (15%) (Table 2). Palpitations were reported by 13% of patients, peripheral edema by 12% and 7% of patients had atypical chest pain (Table 2).

**Table 2:** Cardiac symptoms and laboratory findings of patients with Wilson Disease

Questionnaire cardiac-related symptoms	WD (n = 76)	WD-neuro^−^ (n = 43)	WD-neuro^+^ (n = 33)^a^	p
Dyspnea	12%	5%	23%	< 0.05
NYHA class	1 (1–1)	1 (1–1)	1 (1–2)	0.06
Chest pain (atypical)	7%	2%	13%	0.07
Peripheral edema	12%	9%	16%	0.38
Palpitations	13%	14%	13%	0.90
Dizziness	24%	16%	35%	0.06
Loss of consciousness	15%	10%	29%	0.08
Laboratory Values				
NT-proBNP (< 125 ng/l)	113 ± 121	122 ± 138	100 ± 94	0.53
Creatinine (0.5–0.9 mg/dl)	0.7 ± 0.1	0.7 ± 0.2	0.7 ± 0.1	0.41
eGFR (> 60 ml/min/1.73m^2^)	114 ± 16	114 ± 16	113 ± 16	0.81
Urea (< 45 mg/dl)	28 ± 8	27 ± 7	29 ± 8	0.25
AST (< 37U/l)	37 ± 19	37 ± 20	36 ± 16.3	0.72
ALT (< 35U/l)	53 ± 44	57 ± 44	48 ± 44	0.38
LDH (< 264U/l)	208 ± 45	208 ± 46	208 ± 465.7	0.96
ALP (55–105U/l)	93 ± 35	92 ± 39	95 ± 30	0.74
Total bilirubin (< 1 mg/dl)	0.97 ± 0.7	0.97 ± 0.6	0.98 ± 0.9	0.96
Direct bilirubin (< 0.3 mg/dl)	0.3 ± 0.2	0.3 ± 0.2	0.3 ± 0.2	0.63
Iron (12–27 µmol/l)	16 ± 7	16 ± 8	15 ± 6	0.46
Ferritin (20–120 µg/l)	153 ± 152	152 ± 153	154 ± 153	0.98
Ceruloplasmin (0.2–0.6 ng/l)	0.1 ± 0.05	0.1 ± 0.05	0.1 ± 0.05	0.99
Copper, serum levels (12–24 µmol/l)	5.6 ± 3.5	5.7 ± 3.6	5.6 ± 3.3	0.87
Zinc, serum levels (9–18 µmol/l)	16 ± 7	17 ± 7	15 ± 6	0.26
Non-ceruloplasmin-bound copper (µmol/l)	1.8 ± 1.5	1.8 ± 1.4	1.8 ± 1.8	0.97
Copper, urine levels (0.06–1.26 µmol/l)	1.5 (0.8–2.9)	1.3 (0.6–2.7)	1.5 (0.9–5.2)	0.24
Copper, 24-h urine (< 0.94 µmol/d)	2.6 (1.3–6.0)	2.60 (1.0–5.3)	2.9 (1.3–6.3)	0.36
Zinc, urine levels (2.8–13.0 µmol/l)	14.1 ± 13.2	11.9 ± 11.0	17.0 ± 15.3	0.09
Zinc, 24-h urine (2.3–18.4 µmol/d)	25.6 ± 28.1	23.5 ± 21.3	28.3 ± 35.1	0.48

Cardiac-related symptoms according to questionnaire and laboratory findings of Wilson Disease (WD) patients, WD patients without (WD-neuro^**−**^) and with neurological symptoms (WD-neuro^**+**^): eGFR, estimated glomerular filtration rate; AST, aspartate transaminase; ALT, alanine transaminase; LDH, lactate dehydrogenase; ALP, alkaline phosphatase; NT-proBNP, N-terminal pro B-type Natriuretic Peptide. Values are mean ± SD, median (interquartile range) or n (%). Differences between groups were calculated using t-test or Mann–Whitney U test

^a^Neurological symptoms: tremor (n = 17), dysarthria (n = 12), difficulty swallowing (n = 8), autonomic dysfunction (n = 8), unsteady gait (n = 8), dysgraphia (n = 7), ataxia (n = 6), bradykinesia (n = 6), dystonia (n = 5), rigor (n = 2), disturbance of coordination (n = 1), impaired balance (n = 1) and chorea (n = 1)

WD-neuro^**+**^ patients presented with the following neurological symptoms; tremor (52%), dysarthria (36%), difficulty swallowing (24%), autonomic dysfunction (24%), unsteady gait (24%), dysgraphia (21%), ataxia (18%), bradykinesia (18%), dystonia (15%), rigor (6%), disturbance of coordination (3%), impaired balance (3%) and chorea (3%). The median UWDRS score of WD-neuro^+^ patients was 5.5 (2–12).

Compared with WD-neuro^−^ patients, WD-neuro^**+**^ patients more often reported dyspnea at rest or on exertion (p < 0.05, Table 2) and stated impaired mobility (p < 0.05, Additional file 1: Table S1).

Additionally, significantly more WD-neuro^**+**^ patients stated impaired mobility (p < 0.05, Additional file 1: Table S1). They also showed a non-significant trend towards more atypical chest pain, dizziness and loss of consciousness (p = 0.07, p = 0.06 and p = 0.08 respectively, Table 2).

The mean N terminal pro-hormone brain natriuretic peptide (NT-proBNP) was not elevated. However, thirteen WD patients had elevated NT-proBNP levels (range 134–697 ng/l). Serum creatinine and urea were within normal range. WD patients showed reduced ceruloplasmin and serum copper levels and elevated transaminases, 24-h urine copper and zinc levels (Table 2).

There were no significant differences regarding laboratory values concerning NT-proBNP, kidney and liver function, copper and zinc levels in serum or urine and prescribed medication between WD-neuro^**+**^ and WD-neuro^**−**^ patients (Table 2 and Additional file 1: Table S1).

ECG abnormalities were present in 45% of WD patients; sinus tachycardia (n = 2), sinus bradycardia (n = 1), first degree AV block (n = 1), inverted P wave (n = 2), P enlargement (n = 1), QRS complex prolongation > 100 ms (n = 19), left bundle branch block (n = 1), ST elevation (n = 3), peaked T wave (n = 3) and inverted T wave (n = 7). There were no significant differences in the occurrence of ECG abnormalities between the WD subgroups (Additional file 1: Table S1).

### Cardiac manifestation

There were no significant differences in LV or right ventricular (RV) ejection fraction (EF) or indexed LV and RV volumes between WD patients and healthy controls (Table 3). However, four WD-neuro^+^ patients (5.3%) had a reduced LVEF compared to age- and gender-matched healthy controls. The subjects had no cine CMR evidence of regional dysfunction or LGE indicating absence of occult coronary artery disease.

**Table 3:** Cardiac morphology and function of patients with Wilson Disease

	WD	Controls	p	WD-neuro^−^	WD-neuro^+^	p
CMR Measurements	n = 76	n = 76		n = 43	n = 33	
LV EDV indexed (ml/m^2^)	81 ± 13	80 ± 11	0.57	82 ± 13	79 ± 14	0.41
LVEF (%)	63 ± 5	64 ± 5	0.27	64 ± 4	62 ± 6	0.78
LV SV indexed (ml/m^2^)	51 ± 9	51 ± 7	0.98	52 ± 9	49 ± 9	0.20
LV- mass (g)	105 ± 25	106 ± 28	0.75	101 ± 23	110 ± 26	0.12
Septum (mm)	9 (8–10)	8.5 (8–10)	< 0.05	9 (8–10)	10 (9–11)	0.12
Inferolateral wall (mm)	6 (6–7)	6 (5–7)	0.40	6 (5–7)	7 (6–7)	< 0.05
RV EDV indexed (ml/m^2^)	79 ± 15	82 ± 13	0.57	78 ± 13	79 ± 16	0.76
RVEF (%)	66 ± 7	64 ± 7	0.29	67 ± 6	64 ± 8	< 0.05
RV SV indexed (ml/m^2^)	51 ± 9	51 ± 7	0.84	52 ± 9	50 ± 9	0.28
Strain						
LV-GLS (%)	− 19.6 ± 2.2	− 20.3 ± 1.5	0.09	− 20.0 ± 2.2	− 19.1 ± 2.1	0.09
LV-GCS (%)	− 19.7 ± 2.6	− 21.0 ± 1.3	< 0.01	− 20.5 ± 2.1	− 18.7 ± 2.9	< 0.01
RV-GLS (%)	− 20.5 ± 2.2	− 21.0 ± 1.7	0.18	− 20.6 ± 2.1	− 20.3 ± 2.3	0.61
RV-GCS (%)	− 18.5 ± 2.4	− 19.2 ± 1.5	0.10	− 18.9 ± 1.9	− 18 ± 2.8	0.12

Cardiac Magnetic Resonance (CMR) Measurements of Wilson Disease (WD) patients compared to Controls and WD patients without (WD-neuro^**−**^) and with neurological symptoms (WD-neuro^**+**^): LV, left ventricle; EDV, end-diastolic volume; EF, ejection fraction; SV, stroke volume; CO, cardiac output; MAPSE, mitral annular plane systolic excursion; LA, left atrium; RV, right ventricle; TAPSE, tricuspid annular plane systolic excursion; GLS, global longitudinal strain; GCS, global circumferential strain: Values are mean ± standard deviation or median (interquartile range). Differences between groups were calculated using t-test or Mann–Whitney U test

Two of these patients (60-year-old female and 34-year-old male) presented with dyspnea on exertion (NYHA 2) and one patient with an elevated NT-proBNP (333 ng/l), corresponding to a HF prevalence of 2.6% in WD patients, all with a neurological manifestation. Additionally, RVEF was significantly reduced in WD-neuro^**+**^ patients compared to WD-neuro^**−**^ patients (p < 0.05, Table 3).

**Fig. 2:**
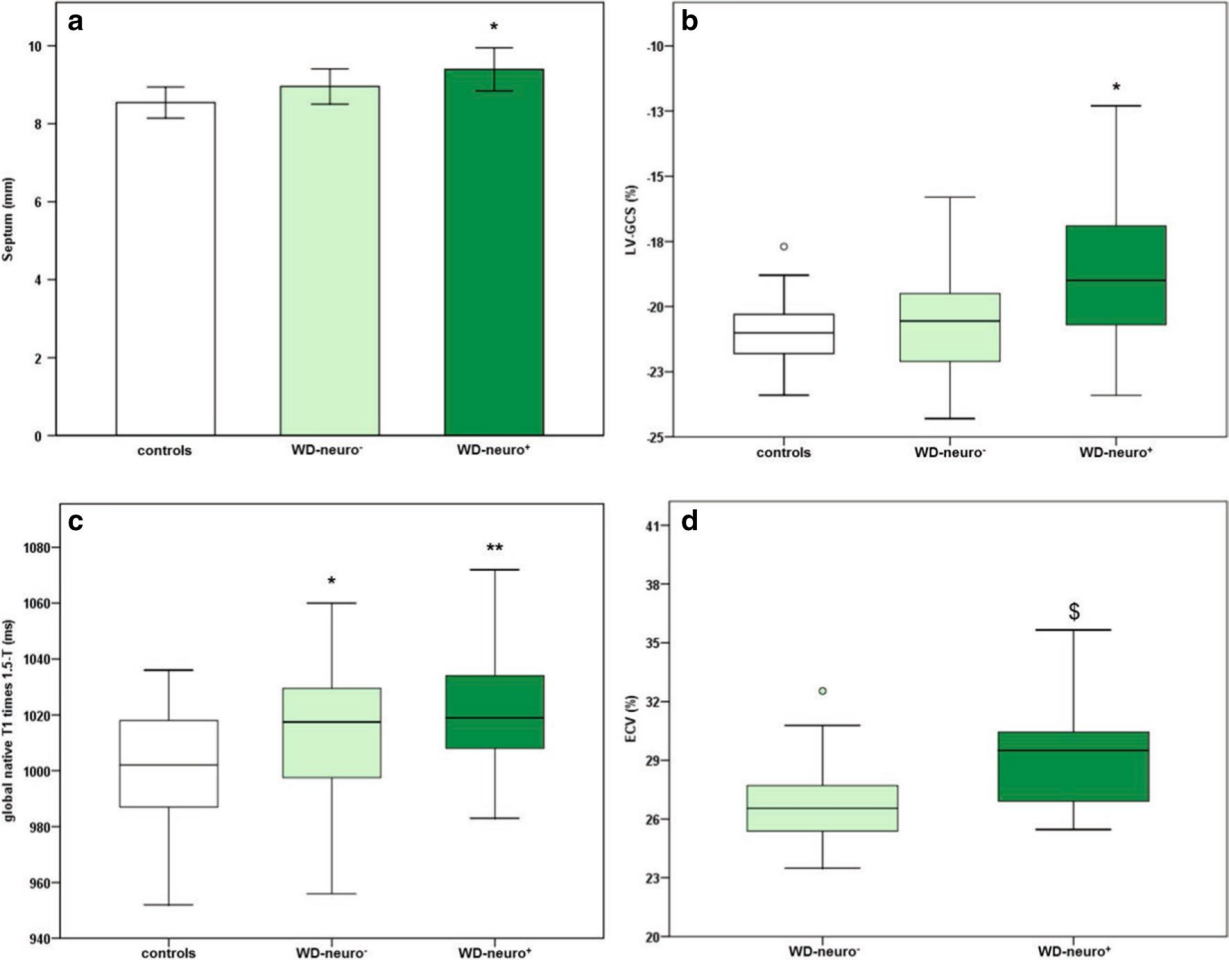
Cardiac structural and functional changes in patients with Wilson Disease with and without neurological symptoms. Comparison of Wilson Disease (WD) patients without neurological symptoms (WD-neuro−), with neurological symptoms (WD-neuro**+**) and healthy controls: Septum (mm) (**a**); Left ventricular global circumferential strain (LV-GCS (%)) (**b**), global native T1 time (ms) (**c**) and extracellular volume fraction (ECV) (%) (**d**) in 1.5-T CMR. Differences between all three groups were calculated using 2-way ANOVA or Kruskal–Wallis test, * p < 0.05 vs. controls; **p < 0.01 vs. controls; $p < 0.05 vs. WD neuro− (t-test)

**Fig. 3:**
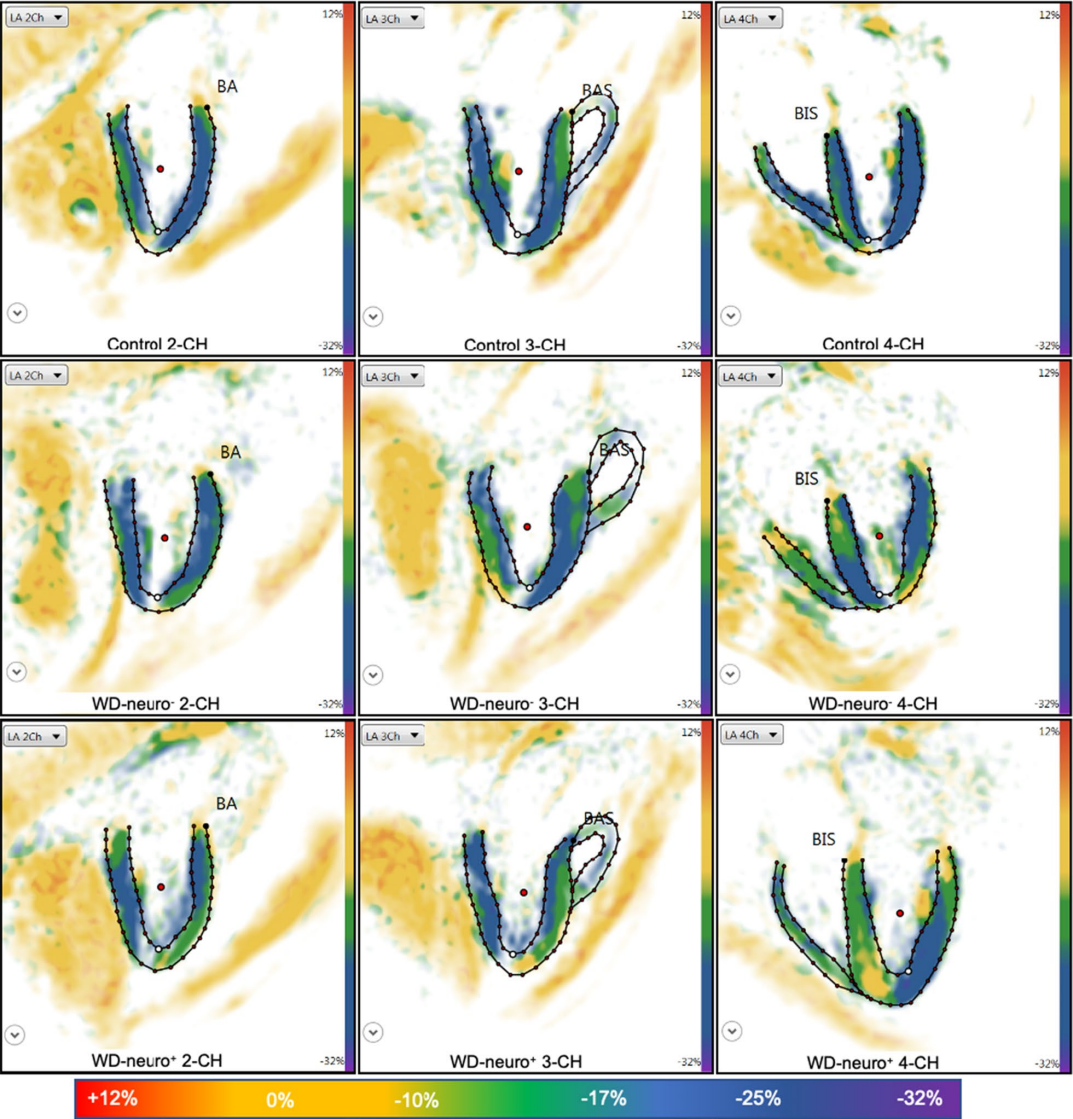
Images of left ventricular global circumferential strain in patients with Wilson. Disease with and without neurological symptoms. Images of strain using fSENC: left ventricular (LV) two- (2-CH), three- (3-CH) and four- (4-CH) chamber views at end-systolic phase used to calculate LV global circumferential strain (GCS). Upper row—controls (male, GCS − 21.5%), middle row—Wilson Disease (WD) patients without neurological symptoms (WD-neuro−) (male, GCS − 20.5%), lower row—WD patients with neurological symptoms (WD-neuro+) (male, GCS − 16.8%). Color maps range from violet indicating strain up to − 32% to red indicating a severely reduced strain (+ 12%) (as shown in color scale)

WD patients demonstrated significantly reduced LV-GCS compared to controls (p < 0.01, Table 3, Figs. 2b,  3) and significantly higher numbers of dysfunctional segments with reduced strain compared to controls (p < 0.001, Table S2). Furthermore, WD-neuro^**+**^ patients showed significantly reduced LV-GCS compared to WD-neuro^**−**^ patients (p < 0.01, Table 3, Figs. 2b and 3). LV-GCS was weakly correlated with urine copper levels (r = 0.278, r^2^ = 0.077, p < 0.05). There were however no significant correlations between copper levels in 24 h urine collection or copper levels in serum and LV-GCS. LV-GLS was similar between controls and WD patients and betwen the WD-neuro+ and WD-neuro- patients (Table 3, Fig. 5).

**Fig. 4:**
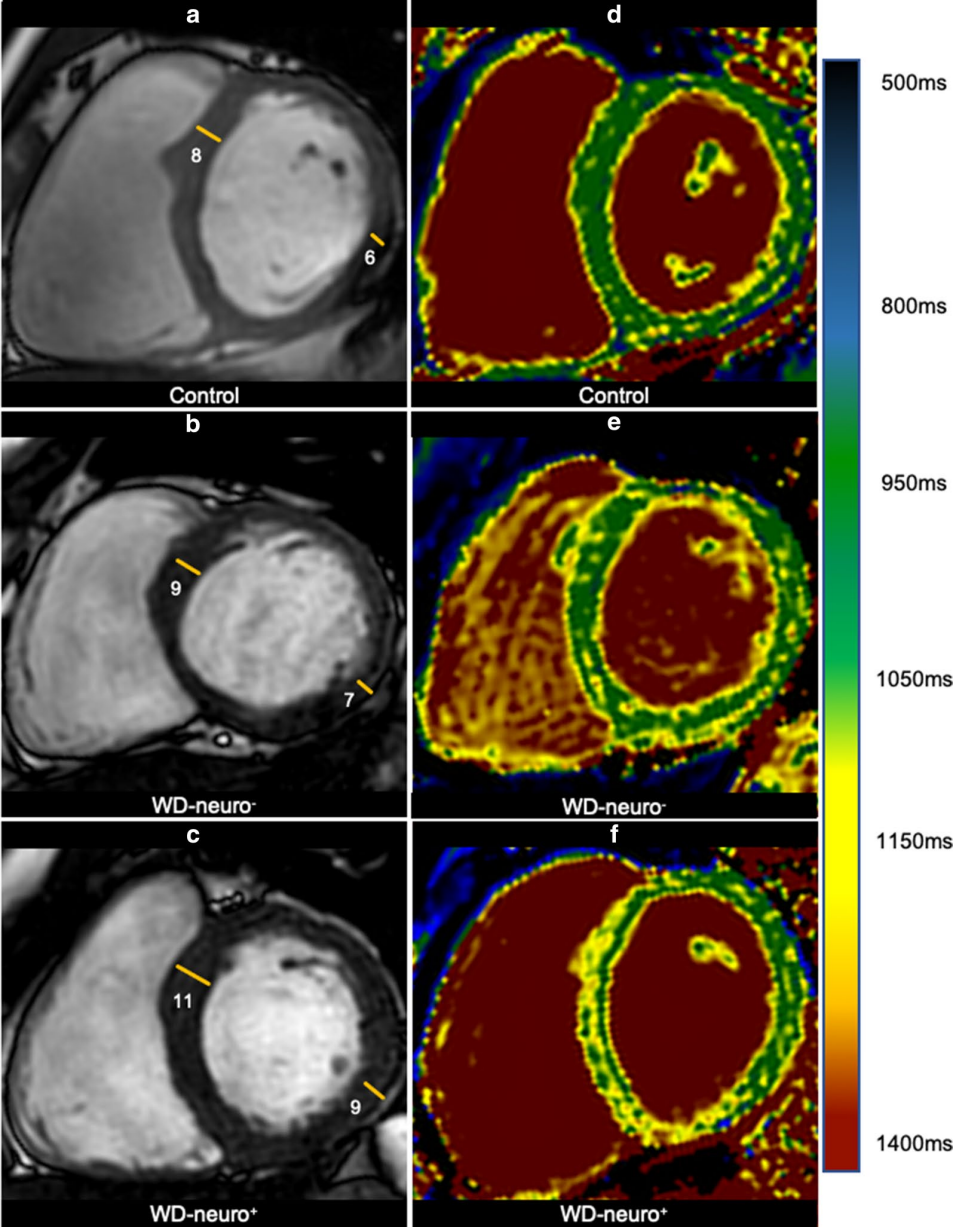
Cine images and native T1 MOLLI in patients with Wilson Disease (WD) with and without neurological symptoms. Images of left ventricular in Wilson Disease (WD) patients: controls (**a**) (male, septum 8 mm, lateral wall 6 mm), WD patients without neurological symptoms (WD-neuro−) (**b**) (male, septum 9 mm, lateral wall 7 mm) and WD patients with neurological symptoms (WD-neuro+) (male, septum 11 mm, lateral wall 9 mm) (**c**). Images of native T1 times in controls (**d**) (female, global native T1 times 1014 ms), WD-neuro− (**e**) (female, global native T1 times 1055 ms) and WD-neuro+ (**f**) (male, global native T1 times 1070 ms). Colors (1.5 T MRI); color scale displays T1 values (ms)

Additionally, WD patients showed a small yet significant increased thickness of the interventricular septum when compared to controls (p < 0.05, Table 3, Figs. 2a,  4a–c). WD-neuro^**+**^ patients had slightly thicker lateral walls compared to WD-neuro^**−**^ patients (p < 0.05, Table 3, Figs. 2a and  4a–c).

**Fig. 5:**
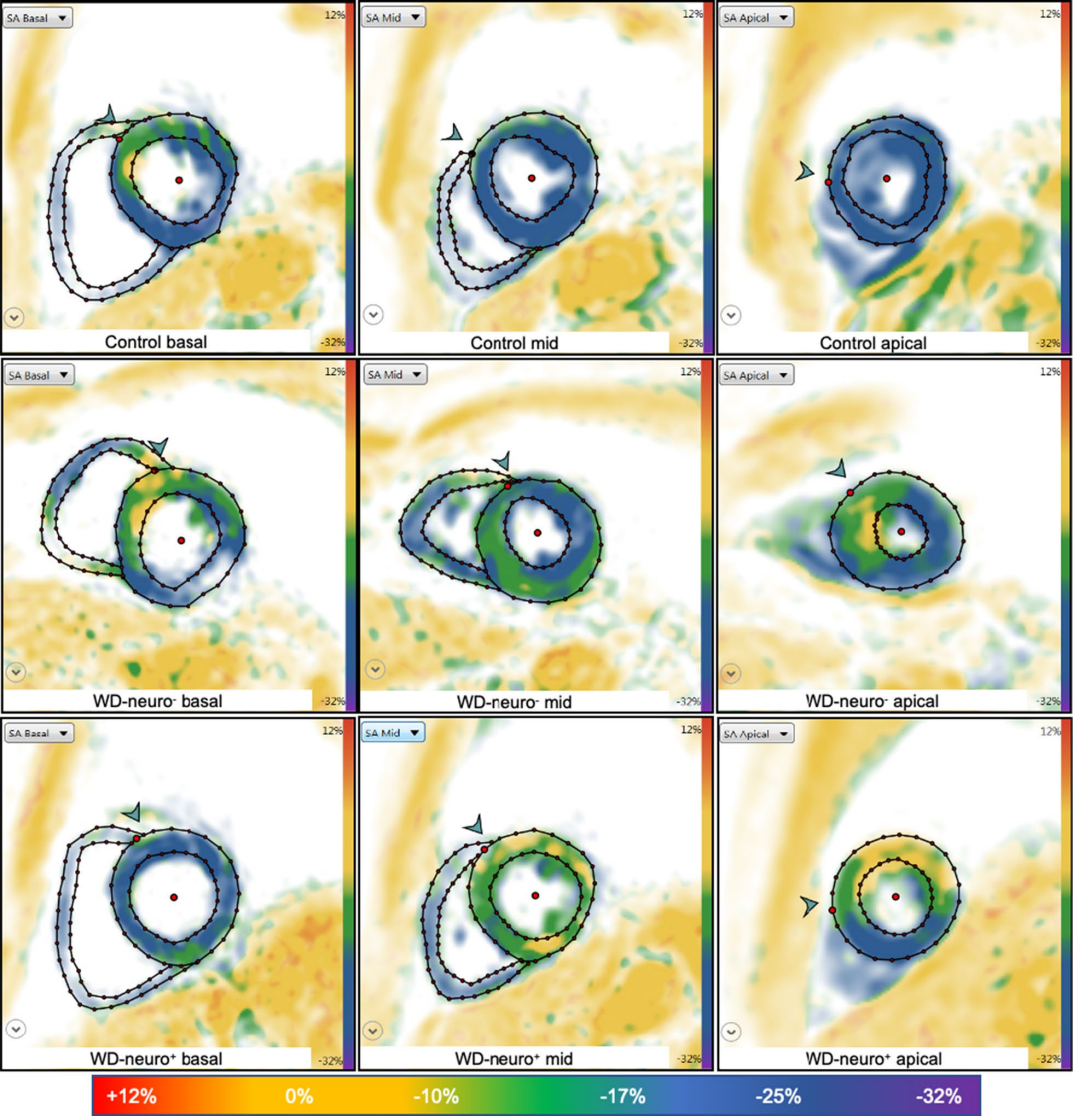
Images of left ventricular global longitudinal strain in patients with Wilson Disease (WD) with and without neurological symptoms. Images of strain using fSENC: left ventricular (LV) short-axis views at LV basal, mid-ventricular (mid) and apical level used to calculate global LV longitudinal strain (GLS). Upper row—controls (male, GLS − 21.8%), middle row—Wilson Disease (WD) patients without neurological symptoms (WD-neuro−) (male, GLS − 17.3%), lower row—WD patients with neurological symptoms (WD-neuro+) (male, GLS − 16.0%). Color maps range from violet indicating strain up to − 32% to red indicating a severely reduced strain (+ 12%) (as shown in color scale)

**Fig. 6:**
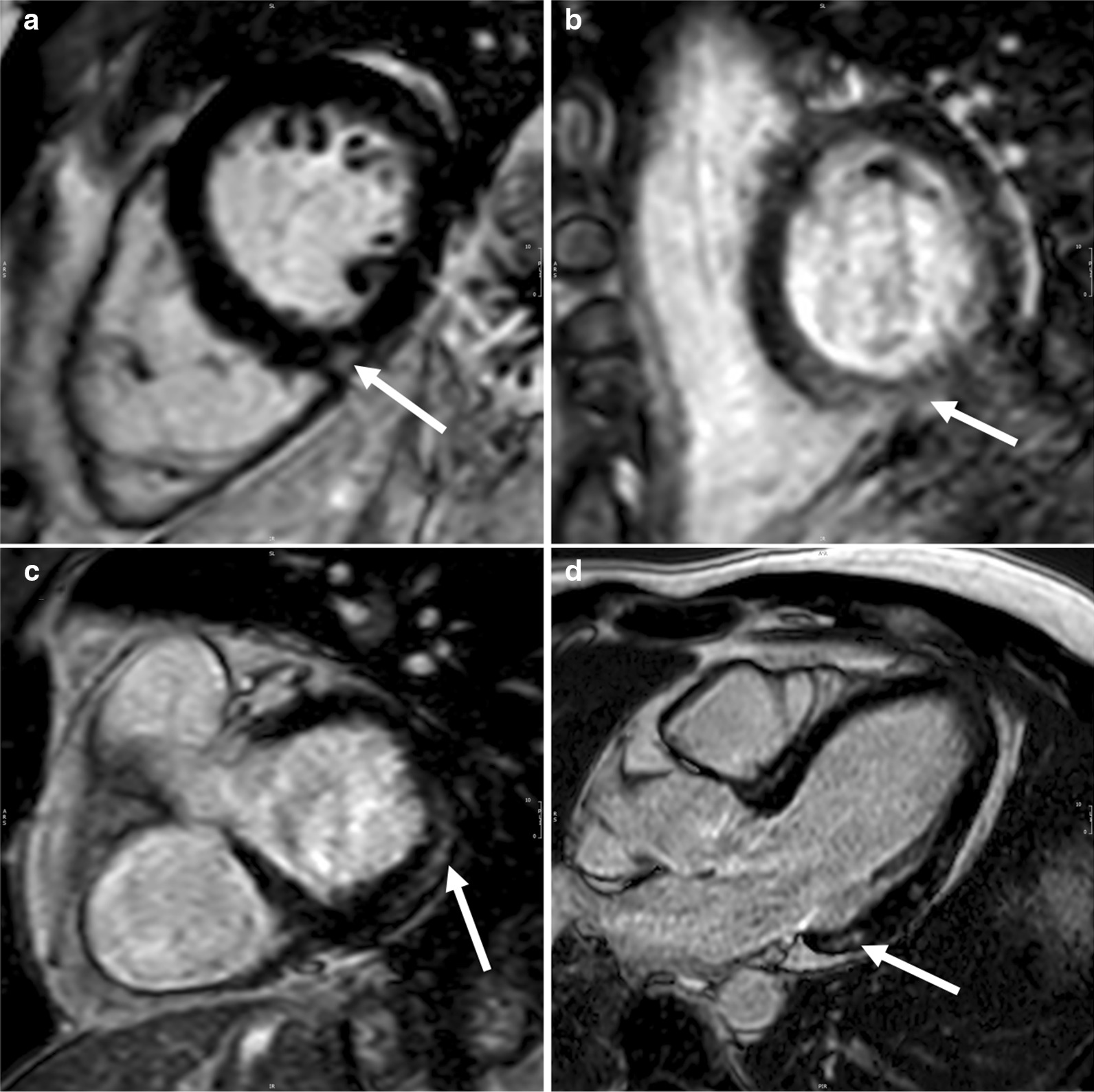
Representative images of late gadolinium enhancement (LGE) in patients with Wilson Disease (WD) with and without neurological symptoms. Representative images of LGE results in WD patients at the inferior insertion point of the right ventricular free wall (**a**), as mid-wall sign (**b**) and subepicardial inferolateral (**c**, **d**)

**Fig. 7:**
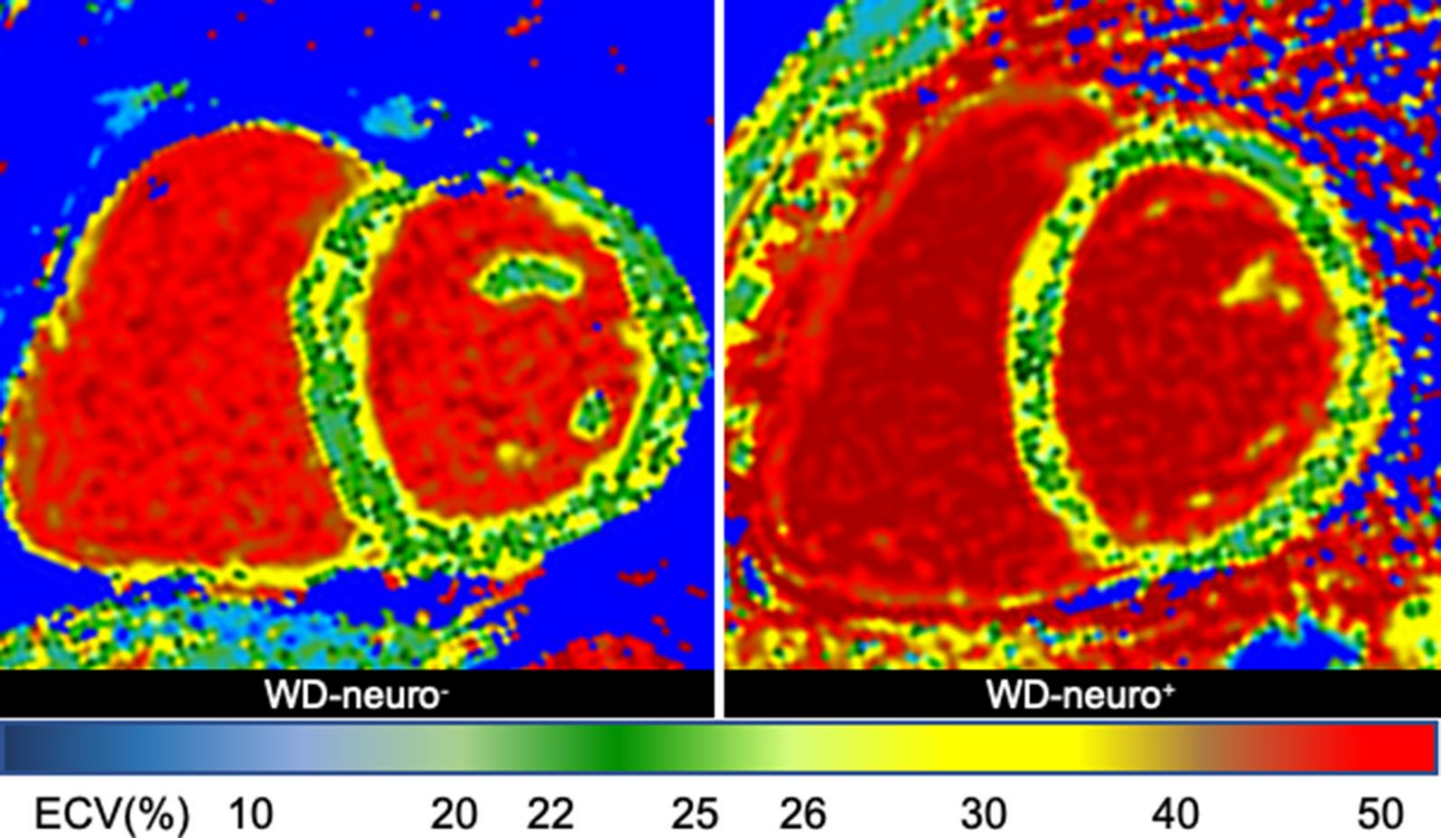
Representative images of extracellular volume fraction (ECV) in patients with Wilson Disease (WD) with and without neurological symptoms. Representative images of extracellular volume (ECV%) in WD patients without (WD neuro−) (male, ECV = 23.5%) (**a**) and WD patients with neurological symptoms (WD-neuro+) (female, ECV = 30.4%) (**b**). Color maps range from blue indicating ECV 1–10% to red indicating severely elevated ECV up to 50% (as shown in color scale)

Global, basal and medial native T1 were significantly higher in WD patients compared to healthy controls (p < 0.01, Table 4, Figs. 2c and  4d–f, 5. Additionally, WD patients showed significantly more LGE compared to controls (p < 0.001, Table 4 and Fig. 6). Thirteen WD patients showed non-ischemic LGE at the inferior insertion point of the RV free wall, eight WD patients at the interventricular septum (midwall) and five subepicardial at the inferolateral wall. WD patients demonstrated an ECV of 27.8 ± 3.0 compared to reference values from the literature (25.0 ± 4.0) [32], indicating increased myocardial fibrosis in WD patients. ECV was significantly higher in WD-neuro^**+**^ patients compared to WD-neuro^**−**^ patients (p < 0.01, Table 4, Figs. 2d and  7). Myocardial fibrosis was increased in WD neuro^+^ compared to WD-neuro^−^ patients. There was no correlation between copper levels in urine, in 24 h urine collection or in serum and native T1 times or ECV (data not shown). T2 times, which indicated myocardial edema and T2* times were within normal range and no significant differences between the two groups were identified (Table 4). Local reference values for T1 and T2 are 997 ± 21 ms and 50.5 ± 3.7 ms, respectively. There was no significant correlation between cardiac findings and the Leipzig score. However, there was a moderate correlation [33] between the UWDRS score and ECV (r = 0.644, r^2^ = 0.415, p < 0.05).

**Table 4:** Myocardial tissue characterization of patients with Wilson Disease

	WD	Healthy controls	p	WD-neuro^−^	WD-neuro^+^	p
	n = 76	n = 76		n = 43	n = 33	
Native T1						
Global (ms)	1017 ± 24	1001 ± 23	< 0.001	1014 ± 25	1022 ± 22	0.17
Basal (ms)	1019 ± 24	1003 ± 25	< 0.01	1017 ± 25	1023 ± 22	0.32
Medial (ms)	1016 ± 30	997 ± 24	< 0.001	1011 ± 28	1023 ± 31	0.10
T2 (ms)						
Global (ms)	51.8 ± 2.2	50.9 ± 1.7	0.08	51.5 ± 2.0	52.2 ± 2.3	0.15
Basal (ms)	50.9 ± 2.7	49.8 ± 1.9	0.10	50.7 ± 2.2	51.1 ± 3.2	0.54
Medial (ms)	51.7 ± 2.6	50.6 ± 2.1	0.07	51.8 ± 2.9	51.6 ± 2.3	0.82
T2^a^ (ms)						
Basal	36.3 ± 3.0	34.9 ± 1.3	0.16	36.9 ± 3.0	35.4 ± 2.9	0.08
Basal septal	37.4 ± 3.0	37.6 ± 1.4	0.83	37.1 ± 2.9	37.9 ± 3.2	0.35
ECV (%)		Reference value^a^				
	27.8 ± 3.0	25.0 ± 4.0		26.7 ± 2.8	29.3 ± 2.6	< 0.01
LGE performed						
Non-ischemic LGE present (%)	26	0	< 0.001	12 (28)	14 (42)	0.10
RVIP (%)	13	0	–	5 (12)	8 (24)	0.15
Midwall (%)	8	0	–	6 (14)	2 (6)	0.27
Subepicardial inferolateral (%)	5	0	–	1 (2)	4 (12)	0.09

Comparison of native T1, T2, T2*, ECV and late gadolinium enhancement (LGE) between Wilson Disease (WD) Patients and healthy controls and between WD Patients without (WD-neuro^**−**^) and with neurological symptoms (WD-neuro^**+**^): RVIP, inferior insertion point of the right ventricular free wall; midwall, interventricular septum: Values are mean ± standard deviation. Differences between groups were calculated using t-test

^a^Dabir et al. Reference values for healthy human myocardium using a T1 mapping methodology: results from the International T1 Multicenter cardiovascular magnetic resonance study. J Cardiovasc Magn Reson, 2014. 16(1): p. 69

## Discussion

### Copper-induced cardiac alterations

Our data indicate that WD patients do not present a distinct, clinically relevant cardiac phenotype. Symptomatic HF occurred in only two WD patients (2.6%). WD-neuro^+^ patients suffered more frequently from symptomatic HF compared to WD-neuro^−^. WD however was associated with CMR markers of global and regional myocardial fibrosis. Furthermore, patients with WD showed an impaired LV GCS, that was weakly correlated with urine copper levels.

The reduced LV GCS in WD patients and in particular in WD-neuro^+^ patients may reflect subclinical cardiac impairment. CMR of myocardial strain is increasingly gaining attention as EF may not be sensitive enough to detect subtle changes of myocardial function [34]. Impaired LV GCS has also been identified as an independent predictor of adverse outcome in patients with suspected cardiomyopathy [35]. LV GCS correlated only weakly with urine copper levels. Hence, a direct cardiotoxic effect of copper resulting in subclinical cardiac dysfunction may be possible, but future investigations, including long-term follow-up studies are required to prove this finding.

It has been demonstrated that the GCS is an important predictor for outcome in patients with other storage diseases, such as light-chain amyloidosis [36, 37]. However, the circumferential myocardial function seems to be important for maintaining LV shape and prevents ventricular dilatation [38]. Mordi et al. showed that GCS is an incremental independent prognostic marker for the endpoint major adverse cardiovascular event (MACE) and all-cause mortality, even in a patient cohort with mildly reduced LVEF [39]. Also, in asymptomatic individuals from the Multi-Ethnic Study of Atherosclerosis without any structural heart disease, GCS provides an independent predictor for future HF [40]. The myocardium consists of fibers oriented in the longitudinal and circumferential directions. The circumferential myocardial fibers are predominant, and the circumferential myocardial function is the main determinant of stroke volume [41]. Therefore, the circumferential function might be affected earlier than the longitudinal in WD patients, while both decrease before LVEF reduces.

The genetic defect of ATP7B results in copper accumulation in the entire body particularly however in the liver and in the brain. It causes interstitial fibrosis, mitochondrial enlargement and vasculitis resulting in cirrhosis and neurological disorders [5, 6, 8, 9]. Little is known about the effects of copper on the heart. However, copper has been shown to increase the oxidation of isoproterenol to isoprenochrome in cardiomyocytes [42]. Isoprenochrome is known to impair mitochondrial energy production, induce cell damage and contractile failure in rat hearts [43]. Additionally, Factor et al. showed myocardial fibrosis in autopsies of young WD patients [44]. WD patients in our study revealed increased myocardial native T1 and LGE. In particular, WD neuro^+^ patients had higher native T1 and a significantly increased ECV compared to healthy controls indicating myocardial fibrosis in particular in WD patients with neurological symptoms. The median UWDRS score positively correlated with ECV, indicating that the severity of neurological symptoms correlates with the degree of myocardial fibrosis. However, there was no correlation with the UWDRS or the Leipzig score and other cardiac findings. Additional studies with a larger patient population are necessary to confirm this finding. There were no significant differences in myocardial T2 between WD patients and healthy controls and in the subgroups analysis, which may indicate myocardial edema [45]. Previous studies demonstrated a close correlation between ECV and native MOLLI T1 and the extent of histological interstitial fibrosis [46, 47]. Increased ECV in HF patients with preserved EF was associated with a higher rate of all-cause death [48]. Recently Quick et al. demonstrated in 11 out of 61 WD patients myocardial LGE at the inferior insertion point of the RV free wall and the interventricular septum, similar to our results [49]. However, to our knowledge, our study is the first that could non-invasively quantify myocardial fibrosis using ECV in WD patients and demonstrate a significant difference in WD patients with and without neurological symptoms. Similar to our results, Quick et al. found no differences in LVEF in WD patients compared to controls. Also, RVEF was significantly reduced in their study group, similarly to our WD-neuro^+^ patients. However, their study results also differ compared to ours, such as they included five patients with a myocarditis-like pattern (myocardium to skeletal muscle signal intensity ratio > 2 in T2-weighted CMR images). Elevated T2 signal of the myocardium quantifies myocardial edema [26], indicating that some patients in their study might have suffered from an acute exacerbation compared to ours [49]. Additionally, the colleagues and also others reported LV hypertrophy in WD patients [10–12, 49]. However, LV hypertrophy was not found in our cohort, even though septum and lateral wall of WD patients was increased, in particular in WD-neuro^**+**^ patients. An explanation for these differences might be the young age of our cohort with mainly asymptomatic or mildly symptomatic patients. Additionally, all of our patients were examined as medical treatment was already been implemented and none of the patients suffered from major acute exacerbation of copper exposure.

Consistent with the overall mild phenotypic expression, symptomatic HF was present in only 2.6% of WD patients. Thus, any copper-induced structural alterations were clinically not relevant. WD-neuro^+^ patients more often complained about dyspnea at rest or on exertion, chest pain, dizziness and loss of consciousness in the past than WD-neuro^−^ patients. These symptoms may also result from neuro-muscular rather than from cardiac dysfunction. However, our results indicate a higher prevalence of cardiac alterations in WD neuro^+^ patients, possibly indicating a higher risk cohort.

Besides increased native T1, there were no characteristic patterns in terms of CMR mapping measurements indicating copper-induced myocardial alterations. Specifically, myocardial T2* was not significant altered in WD patients, consistent with the fact that copper as a diamagnetic metal does not significantly affect myocardial relaxation times or signal intensity in CMR images [50].

Currently, it is not clear to what extent copper accumulation in the myocardium may induce injury or adaptive charges. Cardiac symptoms in WD patients may include cardiomyopathies, arrhythmias, autonomic and diastolic dysfunction, HF and even cardiac death [10–14]. A copper-induced cardiomyopathy has been first described by Kuan et al. characterized by ECG abnormalities and autonomic dysfunction. Two out of fifty-three patients died in their study due to ventricular fibrillation or HF [11].

A number of WD patients (5%) in our study suffered from atrial fibrillation and more than half of the WD-neuro^+^ patients presented with additional ECG abnormalities such as sinus tachycardia, sinus bradycardia, first degree AV block, inverted P wave, P enlargement, QRS complex prolongation, left bundle branch block, ST elevation, peaked T wave and inverted T wave. The prevalence of atrial fibrillation in the general Caucasian population is around 3% [51] and is, at least in patients with type 2 diabetes, increased in the presence of non-alcoholic fatty liver disease [52]. The slightly higher prevalence of atrial fibrillation and ECG abnormalities in our cohort may reflect the more severe myocardial involvement in WD-neuro^+^ patients, but may also be due to secondary effects of advanced liver disease. It is known that advanced liver disease is associated with myocardial fibrosis [53].

Another reason why WD-neuro^+^ patients may reveal more cardiac abnormalities such as remodeling with myocardial fibrosis could be due to a more advanced stage of WD once neurological symptoms become apparent [1, 54]. Since initial signs in WD can be subtle, it may take a considerable time until clinical diagnosis is established [1]. Hence, WD-neuro^+^ patients might have been exposed to higher copper levels for a longer period of time compared to WD neuro^−^ patients. This supports the idea of a direct effect of copper on the myocardium in WD. There is also a close association of urine copper levels and reduced myocardial strain. Additionally, WD-neuro^+^ patients have a worse response rate to treatment compared to WD WD-neuro^−^ patients [55]. Neuronal damage in other neurological conditions such as epilepsy and stroke has been shown to impact cardiac function via a brain–heart interaction [56, 57]. Autonomic dysfunction is also more common in WD-neuro^+^ patients [12]. Increased autonomic dysfunction in patients with neurological disorders of the brain can lead to cardiac fibrosis via catecholaminergic toxicity [56, 58]. Thus the brain–heart interaction via autonomic dysfunction might be an additional factor resulting in cardiac alterations in WD patients, particularly in those with neurological symptoms.

### Clinical impact

Commonly clinical symptoms and disease progression in WD are driven by the liver and nervous system manifestation while cardiac involvement is less severe [59]. Therefore, during early stages of WD, patients may not need frequent specific screening for cardiac involvement, unless they show symptoms or signs of cardiac disease. However, with disease progression and the development of symptoms or signs of neurological involvement, patients may develop myocardial fibrosis. Because the latter is associated with an overall increased risk for HF and death, dedicated cardiac care is required. In fact, a recent case report on a patient with sudden cardiac death in Wilson Disease, associated with LGE, underscores the role of fibrosis as a potentially critical marker in WD [60].

CMR examinations in suspected WD should focus on function/strain and fibrosis imaging. Both indexes can be assessed without the use of gadolinium contrast. fSENC and T1 mapping are highly reproducible and have an excellent (very low) intra- and interobserver variability, thus providing robust data on cardiac function and myocardial fibrosis [15, 47, 61, 62]. Although severe cases of cardiac involvement in WD have been reported previously, those case reports were before improved therapy was widely available [11]. The fact that symptomatic HF only occurred in a minority of patients in our study may also be due to better monitoring and treatment of WD patients at our referral center.

## Limitations

Our study has limitations. Most of the patients presented with stabilized symptoms or were asymptomatic. Furthermore, the young age of our cohort might be a reason for the low incidence of HF compared to other studies. Grandis et al. demonstrated a prevalence of 13.3% of HF in WD patients. However, patients who developed HF in their study had a mean age of 66 ± 18 years and were therefore older than the subjects in our study (mean age 37 ± 14 years). Obviously, cardiac manifestation in WD might be more severe in an untreated and older cohort or in patients with an exacerbation of the disease.

Additionally, even though a correlation between LV GCS and copper levels in urine was identified, this needs to be confirmed in a larger cohort. Follow up studies are necessary to clarify if a reduced LV GCS is a predictor for a worse prognosis in WD patients. Also, no biopsies of the myocardium were performed, which prevents a clear histological correlation of the regions with increased myocardial T1 times, ECV and LGE. While our results show that WD patients, particularly WD-neuro^+^ patients have a subclinical cardiac dysfunction and myocardial fibrosis, prospective studies are required to investigate the development of cardiac impairment, myocardial fibrosis and prognosis of these findings.

## Conclusions

Overall, cardiac alterations of WD patients are mild and many patients do not reveal a clinically relevant heart disease. In the subgroup of patients with symptoms or signs of neurological involvement, patients carry a higher risk for the development of myocardial fibrosis which is an established risk indicator. The clinical and prognostic impact of these findings have to be further elucidated.

## Authors´ contributions

JS contributed to conception and design of the study, collected patient cohort and control group, analyzed and interpreted CMR, ECG, laboratory and clinical data, wrote the manuscript and revised it critically. IM collect patient cohort, interpreted CMR, clinical and laboratory data of patients, and revised the manuscript critically. JH collected patient cohort and analyzed and interpreted CMR, ECG, laboratory and clinical data of patients. MHC analyzed and interpreted CMR fSENC data. AO analyzed and interpreted CMR mapping data. OP collected and analyzed the control group regarding CMR data. JR contributed to design of the study and discussion of data. FA contributed to design of the study and discussion of data. KH collected and analyzed the control group regarding CMR data. MMH collected clinical data of control group. EG collected clinical data of control group. MGF contributed to conception and design of the study, drafted the manuscript and revised it critically. UM contributed to conception and design of the study. KHW contributed in conception and design of the study and revised the manuscript critically. HAK interpreted data and revised the manuscript critically. MO contributed significantly to conception and design of the study, interpreted CMR, ECG, laboratory and clinical data of patients, drafted the manuscript and revised it critically. All authors read and approved the final manuscript.

## Supplementary Information

**Additional file 1: Table S1.** Impaired mobility, additional laboratory and ECG findings of Wilson Disease patients. **Table S2.** Cardiac morphology and function of Wilson Disease patients.

## Data Availability

The datasets used and analysed during the current study are available from the corresponding author on reasonable request.

## Abbreviations

BSA: Body-surface area
CMR: Cardiovascular magnetic resonance
ECG: Electrocardiogram
ECV: Extracellular volume fraction
EDV: End-diastolic volume
EF: Ejection fraction
GCS: Global circumferential strain
GLS: Global longitudinal strain
HF: Heart failure
IQR: Interquartile range
LGE: Late gadolinium enhancement
LV: Left ventricle/left ventricular
MOLLI: Modified look-locker inversion recovery
NT-pro-BNP: N-terminal pro-hormone brain natriuretic peptide
NYHA: New York Heart Association
RV: Right ventricle/right ventricular
SENC: Strain encoding
SV: Stroke volume
UWDRS: Unified Wilson’s Disease Rating Scale
WD: Wilson Disease
WD-neuro^+^: Wilson Disease with predominant neurology manifestations
WD-neuro^−^: Wilson Disease without neurologic manifestations

## Declarations

### Authors´ information

Parts of this work were used for the MD thesis of JH.
