# Supplementary material for: The impact of Wilson disease on myocardial tissue and function: a cardiovascular magnetic resonance study
Source: J Cardiovasc Magn Reson. 2021 Jun 24;23:84. doi: 10.1186/s12968-021-00760-1 (PMC8223377; doi:10.1186/s12968-021-00760-1)
Supplement: Supplementary file 1 — Additional file 1: Table S1. Impaired mobility, additional laboratory and ECG findings of Wilson Disease patients. Table S2. Cardiac morphology and function of Wilson Disease patients. [file 12968_2021_760_MOESM1_ESM.docx]

**Supplementary Materials**

**Full Title: The Impact of Wilson Disease on Myocardial Tissue and Function – A Cardiovascular MR Study**

**Authors:**

Janek Salatzki, MD,^a,b^ Isabelle Mohr, MD,^c^ Jannick Heins,^a^ Mert H. Cerci,^a^ Andreas Ochs, MD,^a,b^ Oliver Paul,^a^ Johannes Riffel, MD,^a,b^ Florian André, MD,^a,b^ Kristóf Hirschberg MD,^d^ Matthias Müller-Hennessen, MD,^a,b^ Evangelos Giannitsis, MD,^a,d^ Matthias G. Friedrich, MD,^a,b,e^ Uta Merle, MD,^c^ Karl Heinz Weiss, MD,^c,f^ Hugo A. Katus, MD,^a,b^ Marco Ochs, MD,^a,b^

**Affiliations:**

^a^Department of Cardiology, Angiology and Pneumology, Heidelberg University Hospital, Heidelberg, Germany (JS, JH, MHC, AO, OP, JR, FA, MMH, EG, MGH, HAK, MO)

^b^DZHK (German Centre for Cardiovascular Research), Partner site Heidelberg, Germany (JS, AO, JR, FA, MMH, EG, MGH, HAK, MO)

^c^Department of Gastroenterology, Heidelberg University Hospital, Heidelberg, Germany (IM, UM, KHW)

^d^Semmelweis University Heart and Vascular Center, Budapest, Hungary (KH)

^e^Division of Cardiology, Departments of Medicine and Diagnostic Radiology, Mc-Gill University Health Centre, Montreal, Canada (MGF)

^f^Department of Internal Medicine, Salem Medical Center, Heidelberg, Germany (KHW)

**Disclosures:**

The project received research support (funding) from „Eva Luise und Horst Koehler Stiftung fuer Menschen mit Seltenen Erkrankungen“.

**Correspondence:**

Janek Salatzki, Department of Cardiology, Angiology and Pneumology, Heidelberg University Hospital, Heidelberg, Germany

e-mail: [janek.salatzki@med.uni-heidelberg.de](mailto:janek.salatzki@med.uni-heidelberg.de)

**Methods**

**Cardiovascular magnetic resonance image acquisition protocol and post-processing**

Following localizing scans, cine long axis 2-, 3- and 4-chamber views as well as short axis cine images covering the whole left ventricle (LV) from the anulus of the atrioventricular valves to the apex (8 mm slice thickness, no gap between each slice) were obtained using a breath-hold, segmented-k-space balanced steady-state free precession sequence (bSSFP) employing retrospective ECG or pulse oximetric gating with 35 phases per cardiac cycles. Scan parameters were: repetition time (TR) 2.8ms; echo time (TE) 1.4ms; flip angle (FA) 60°; with a breath-hold time of 7–10 s per image and prospective gating. Data were analyzed using the cvi^42^ software (Version 5.6.6, Circle Cardiovascular Imaging Inc., Calgary, Alberta, Canada) as semi-automatic software for volumetric analysis. Ventricular volumes, ejection fraction of LV and right ventricle (RV) and LV myocardial mass were acquired in short axis stacks by manually tracing epi- and endocardial borders, excluding papillary muscles from the myocardium.

**Myocardial tissue characterization using Mapping and LGE**

In order to measure pre-contrast T1 times, modified Look-Locker inversion recovery (MOLLI) sequence (5 s(3 s)3 s variant) with following parameters was used: TR 2.3ms, TE 1.06ms, FA 35°. Post-contrast MOLLI sequence (4s(1 s)3 s(1s)2s) variant) used the following specific parameters: TR 2.4ms, TE 1.08ms, FA 35°. Myocardial T2 times were measured using the following parameters: TR was one RR interval, 9 echoes, TE 18ms, TR, FA 90°. T2* quantification used the following parameters: 3 short-axis slices; TR 22ms TE1 1.31ms, delta TE 1.4, FA 20°; acquisition matrix=196 × 140; slice thickness=8 mm.

After visual inspection of all segments and exclusion of those segments with evidence of artefact, pre- and post-contrast T1 and T2 maps were generated using cvi^42^ software (Version 5.6.6, Circle Cardiovascular Imaging Inc.). Endocardial and epicardial borders were defined manually, using an offset of 10% to avoid partial-volume effects in the subendocardial and subepicardial layers. The global T1 and T2-were calculated as a mean of all segments with respect to the segments area. T1 values for blood were detected by manually drawing a region of interest in the LV cavity. If more than two segments in T1, T2, T2* or extracellular volume fraction (ECV) showed evidence of artefacts, the images were excluded from further analysis. This did not change statistical significance (data not shown).

Basal and medial segments for T1 and T2 were measured using T1 mapping and global T1 and T2 was measured by calculating the mean over all segments.

Late gadolinium enhancement (LGE) images were acquired employing a T1-weighted inversion recovery-prepared fast gradient echo sequence with an optimized inversion time. Regions with LGE were verified in at least one other orthogonal plane and in the same plane being obtained as a second image after changing the direction of readout.

**fSENC**

fSENC imaging parameters were: TR 12 ms, TE 0.7 ms, FA 30°, slice thickness 10 mm, field-of-view 256 x 256 mm^2^, and temporal resolution is 39 ms. To cover the ventricles, three long axis (2-, 3- and 4-chamber views) as well as three short axis views (basal, midventricular (mid) and apical LV levels) were acquired. If more than two long or short axis showed relevant evidence of artefacts and reduced image quality, the cases were excluded from further analysis. This did not change statistical significance (data not shown). Longitudinal and circumferential strains are measured in a range between 5% and -30%. The use of negative values is to represent the shortening of muscle associated with contraction.

A 16-segment scheme was used for presenting the longitudinal strain while an 18-segment one was used for the circumferential strain. LV-global longitudinal strain (GLS) was calculated as the average of all 16 segments acquired from the short axis views, and the LV-global circumferential strain (GCS) was the average of all 18 segments acquired from the long-axis views. As mentioned before, both longitudinal and circumferential strain values are negative as normal cardiac muscles in the ventricles contract in the longitudinal and circumferential directions during systole, and will therefore be reported as such. We will, however, refer to the absolute values throughout the text consistent with most reports in the literature (less “numbers” mean lower strain values and therefore reduced deformation).

**Results**

Table S1 Impaired mobility, additional laboratory and ECG findings of Wilson Disease patients

| **Impaired Mobility** | **WD** (n=76) | **WD-neuro^-^** (n=43) | **WD-neuro^+^** (n=33) | **p** |
| --- | --- | --- | --- | --- |
| Ability climbing stairs |  |  |  | <0.05 |
| ≥3 | 91% | 97% | 79% | <0.05 |
| 1-2 | 7% | 3% | 14% | 0.08 |
| 0 | 2% | 0% | 7% | 0.09 |
| **Laboratory Findings** |  |  |  |  |
| Sodium (135-146mmol/l) | 140 ± 2 | 140 ± 2 | 140 ± 2 | 0.56 |
| Potassium (3.4-4.6mmol/l) | 4.1 ± 0.3 | 4.1 ± 0.3 | 4.0 ± 0.3 | 0.18 |
| Protein (60-80g/l) | 72.0 ± 5.05 | 72.0 ± 5.05 | 71.6 ± 5.7 | 0.73 |
| Serum Albumin (30-50g/l) | 44.8 ± 3.26 | 44.8 ± 3.35 | 44.8 ± 3.19 | 0.98 |
| pH value, urine (6-8) | 6.14 ± 0.91 | 6.00 ± 0.84 | 6.34 ± 0.99 | 0.12 |
| Creatinine, urine levels (24-392mg/dl) | 98.8 ± 69.8 | 92.2 ± 60.7 | 108.9 ± 82.0 | 0.34 |
| Albumin, urine levels (<20mg/dl) | 16.6 ± 34.9 | 20.5 ± 42.9 | 9.6 ± 8.17 | 0.34 |
| RBC (4-5.2/pl) | 4.79 ± 0.44 | 4.83 ± 0.37 | 4.75 ± 0.51 | 0.52 |
| HCT (0.36-0.47l/l) | 0.41 ± 0.03 | 0.41 ± 0.03 | 0.41 ± 0.03 | 0.90 |
| HB (12-15g/dl) | 14.0 ± 1.1 | 14.0 ± 1.1 | 14.0 ± 1.0 | 0.79 |
| MCV (83-97fl) | 86.7 ± 3.73 | 86.5 ± 3.21 | 87.0 ± 4.35 | 0.58 |
| MCH (27-33pg) | 29.4 ± 1.55 | 29.4 ± 1.51 | 29.5 ± 1.62 | 0.65 |
| MCHC (30-36g/dl) | 34.1 ± 1.38 | 34.2 ± 1.60 | 33.9 ± 1.01 | 0.31 |
| RDW (12.9-18.7%) | 13.7 ± 1.40 | 13.6 ± 1.36 | 13.9 ± 1.45 | 0.48 |
| **ECG** |  |  |  |  |
| Heart Rate (bpm) | 66 ± 12 | 67 ± 13 | 66 ± 12 | 0.68 |
| PQ interval (ms) | 149 ± 23 | 146 ± 21 | 153 ± 24 | 0.19 |
| QRS complex interval (ms) | 95 ± 11 | 93 ± 9 | 97 ± 13 | 0.10 |
| QT interval (ms) | 386 ± 32 | 387 ± 34 | 385 ± 30 | 0.77 |
| QTc interval (ms) | 396 ± 24 | 398 ± 23 | 394 ± 26 | 0.48 |
| Patients with ECG abnormalities (%) | 34 (45) | 16 (37) | 18 (54) | 0.13 |
| Sinus tachycardia | 2 | 1 | 1 | 0.85 |
| Sinus bradycardia | 1 | 0 | 1 | 0.25 |
| AV-Block I° | 2 | 0 | 2 | 0.10 |
| inverted P wave - Lead II | 2 | 2 | 0 | 0.50 |
| P enlargement - Lead II | 1 | 0 | 1 | 0.25 |
| QRS complex prolongation (>100ms) | 19 | 8 | 11 | 0.14 |
| Left Bundle Branch Block | 1 | 0 | 1 | 0.25 |
| ST elevation | 3 | 1 | 2 | 0.41 |
| peaked T wave | 3 | 1 | 2 | 0.41 |
| inverted T wave | 7 | 3 | 4 | 0.44 |
| **Medication** |  |  |  |  |
| Penicillamine | 35 | 22 | 13 | 0.31 |
| Trientine | 29 | 17 | 12 | 0.78 |
| Zinc | 20 | 11 | 9 | 0.87 |

Impaired mobility asked according to questionnaire and additional laboratory findings of Wilson Disease (WD) patients, WD patients without (WD-neuro**^-^**) and with neurological symptoms (WD-neuro**^+^**): RBC - Red Blood Cells; HCT – Hematocrit; HB – Hemoglobin; MCV - Mean Corpuscular Volume; MCH - Mean Corpuscular Hemoglobin; MCHC - Mean Corpuscular Hemoglobin Concentration; RDW - Red Cell Distribution Width. Electrocardiogram (ECG) findings and medical treatment. Differences between groups were calculated using t-test or chi-squared test.

Table S2 Cardiac morphology and function of Wilson Disease patients

|  | **WD** | **Controls** | **p** | **WD-neuro^-^** | **WD-neuro^+^** | **p** |
| --- | --- | --- | --- | --- | --- | --- |
| **CMR Measurements** | n=76 | n=76 |  | n=43 | n=33 |  |
| LV-ESV indexed (ml/m²) | 29.8 ± 6.4 | 28.7 ± 6.0 | 0.30 | 29.7 ± 5.76 | 29.9 ± 7.2 | 0.89 |
| MAPSE (mm) | 14 (12.5-15) | 14 (12-15) | 0.80 | 14 (13-15) | 13 (12-15) | 0.10 |
| LA Diameter (mm) | 33.5 ± 4.9 | 32.8 ± 5.3 | 0.79 | 33.81 ± 4.67 | 33.1 ± 5.3 | 0.51 |
| RV-ESV indexed (ml/m²) | 27.5 ± 8.7 | 30.2 ± 9.2 | 0.30 | 26.0 ± 7.30 | 29.3 ± 10.1 | 0.10 |
| TAPSE (mm) | 24 (22-28) | 24 (21-28) | 0.58 | 24 (22-28) | 25 (21-29) | 0.57 |
| RA Diameter (mm) | 46.5 ± 5.7 | 46.9 ± 5.3 | 0.65 | 46.4 ± 5.4 | 46.7 ± 6.1 | 0.84 |
| **Strain** |  |  |  |  |  |  |
| No. of dysfunctional segments > -10 | 1 (0-3) | 0 (0-0.5) | <0.001 | 1 (0-2) | 2 (0-4) | <0.05 |
| No. of dysfunctional segments > -17 | 9 (4-13) | 6 (3-9) | <0.01 | 8 (4-11) | 12 (7-14) | <0.01 |

CMR - cardiovascular magnetic resonance; WD – Wilson Disease patients; WD patients without – WD-neuro**^-^** ; WD patients with neurological symptoms – WD-neuro**^+^**; LV – left ventricle; ESV - end-systolic volume; MAPSE – mitral annular plane systolic excursion; LA – left atrium; RV – right ventricle; TAPSE - tricuspid annular plane systolic excursion; RA – right atrium: Values are mean ± standard deviation or median (interquartile range). Differences between groups were calculated using t-test or Mann-Whitney U test.
